# Long-distance temporal quantum ghost imaging over optical fibers

**DOI:** 10.1038/srep26022

**Published:** 2016-05-19

**Authors:** Shuai Dong, Wei Zhang, Yidong Huang, Jiangde Peng

**Affiliations:** 1 Tsinghua National Laboratory for Information Science and Technology, Department of Electronic Engineering, Tsinghua University, Beijing, 100084, China

## Abstract

Since the first quantum ghost imaging (QGI) experiment in 1995, many QGI schemes have been put forward. However, the position-position or momentum-momentum correlation required in these QGI schemes cannot be distributed over optical fibers, which limits their large-scale geographical applications. In this paper, we propose and demonstrate a scheme for long-distance QGI utilizing frequency correlated photon pairs. In this scheme, the frequency correlation is transformed to the correlation between the illuminating position of one photon and the arrival time of the other photon, by which QGI can be realized in the time domain. Since frequency correlation can be preserved when the photon pairs are distributed over optical fibers, this scheme provides a way to realize long-distance QGI over large geographical scale. In the experiment, long-distance QGI over 50 km optical fibers has been demonstrated.

How to realize photonic quantum information applications over long-distance optical fibers is an important topic of quantum engineering, considering the extensive optical fiber links over globe. Quantum information applications realized over optical fibers, can support related, interesting applications over large geographical scale, such as quantum key distribution (QKD)^1^, and quantum teleportation^2^. In this work, we explore the way to realize long distance quantum ghost imaging (QGI) over optical fibers. QGI^3^ has attracted much attention in the last two decades due to its abundance in physics and potential for quantum communication and quantum sensing. Originally, QGI was realized by photon pairs generated by spontaneous parametric down conversion (SPDC) in nonlinear crystals. The momentum-momentum correlation in the photon pairs was utilized in QGI to realize imaging in a nonlocal manner. After that, many ghost imaging schemes have been proposed^4^,^5^,^6^,^7^,^8^,^9^,^10^,^11^,^12^,^13^. The concept of the ghost imaging has been deeply developed “from quantum to classical to computational”^14^. While, the motivation of research on ghost imaging also developed from the interest on fundamental physics^15^,^16^ to applications such as information transmission and sensing. It has been reported that ghost imaging can be used in optical information encryption and transmission^17^. However, it is still a problem how to realize QGI over large geographical scale by techniques compatible with optical communication and networks, since single mode fiber (SMF) used in optical communication does not support the distribution of the position-position or momentum-momentum correlation required in previous QGI schemes.

In recent years, the effect of temporal dispersion on the correlated/entangled photon pairs has attracted much attention and been used in many applications, such as the manipulation of the single photon wave packet^18^, the tunable optical delay between two correlated photons^19^, the measurement of the joint spectral distribution of biphoton states^20^ and non-local dispersion control^21^ and cancellation^22^. In these applications, the arrival time of photons is diffused according to their frequencies. We notice that the photons can also be diffused to different propagation directions according to their frequencies by introducing spatial dispersion on them. If temporal dispersion and spatial dispersion are introduced on the two photons with frequency correlation respectively, the frequency correlation would be transferred to the correlation of arrival time - propagation direction, which can be utilized to realize the QGI in time domain. Hence, we propose and demonstrate a temporal QGI scheme based on frequency correlated photon pairs in this paper. It’s well known that the frequency is a stable degree of freedom (DOF) of photons traveling in optical fibers. The frequency correlation can be preserved when the photon pairs are distributed over optical fibers. Hence, the proposed scheme supports long-distance QGI over optical fibers. To realize the scheme, the frequency correlated photon pairs should be at telecom band, which can be generated by spontaneous four wave mixing (SFWM) processes in optical fibers^23^ or waveguides^24^. The two photons in the pair are sent to Alice and Bob sides, respectively. At Alice side, the photons are spatially dispersed to different directions according to their frequencies by a spatial dispersion component, such as a grating, then illuminate the object on different positions along a line. At Bob side, large temporal dispersion is applied to the idler photons to change their arrival time according to their frequencies when detected by a single photon detector (SPD). To realize long distance QGI, the temporal dispersion could be realized by the transmission fiber sending the idler photons from the source to Bob side. Hence, the frequency correlation in the initial photon pairs is transformed to the correlation of the illuminating positions of the photons at Alice side and the arrival time of the photons at Bob Side. Based on this correlation, the image of the object along the illuminating line can be obtained in time domain by the coincidence measurement, realizing one-dimensional QGI. Two-dimensional imaging can also be realized by step-moving the object, realizing a function of long distance “quantum fax machine” over optical fibers.

## Results

### Principle

Frequency correlated photon pairs can be generated in third order nonlinear waveguide by the spontaneous four wave mixing process^23^,^24^,^25^. Their state can be expressed as


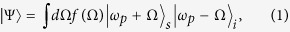


where *ω*_*p*_ is the frequency of the pump light. The indices *s* and *i* indicate the signal photons (with frequency *ω*_*s*_) and idler photons (with frequency *ω*_*i*_), respectively. 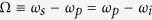 is the frequency detuning of signal photons or idler photons. *f*(Ω) is the spectral amplitude of the biphoton state.

The signal and idler photons are distributed to two parties, named Alice and Bob, over optical fibers. At Alice side, there is an object with a specific reflectivity pattern. The signal photons are firstly dispersed to different directions along a line according to their frequencies by a spatial dispersion component, such as a grating. Then they illuminate the object along a line, which is named as the illuminating line. Signal photons with different frequenciy will arrive at different positions on the object. The signal photons with a specific frequency detuning Ω would arrive at a specific position *x*_Ω_ in the line. The reflectivity pattern along the illuminating line would modulate the spectrum of the reflected signal photons, and also that of the frequency correlated photon pairs, which can be looked as a special form of wave packed reshaping based on energy-time entanglement^26^. Hence, the positive-frequency field operator of the signal photons at Alice side can be expressed as


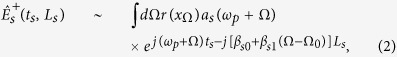


where 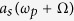 is the annihilation operator of the signal photons at the frequency *ω*_*p*_ + Ω. *t*_*s*_ is the detection time of the signal photons, *L*_*s*_ is the length of the optical fiber between the photon-pair source and the SPD at Alice side. Assuming that *L*_*s*_ is small, the group velocity dispersion (GVD) in the fiber has been neglected. The phase coefficient of the signal photons in the fiber has been expanded in the vicinity of *ω*_*p*_ + Ω_0_ , which is the central frequency of signal photons, as *β*_*s*_(*ω*_*p*_ + Ω) = *β*_*s*0_ + *β*_*s*1_(Ω − Ω_0_).

At Bob side, the idler photons are temporally dispersed before detected by a SPD. The most convenient way to realize the dispersion is utilizing the GVD of the transmission fiber, by which the idler photons are sent to Bob side from the source. If the length of the optical fiber between the photon-pair source and the SPD at Bob side is *L*_*i*_, then the positive-field operator of the idler photons at time *t*_*i*_ can be expressed as


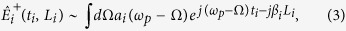


where *a*_*i*_(*ω*_*p*_ − Ω) is the annihilation operator for idler photons at the frequency *ω*_*p*_ − Ω. The phase coefficient of the idler photons in the fiber can be expanded as


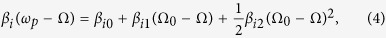


where *ω*_*p*_ − Ω_0_ is the central frequency of idler photons. The term with *β*_*i*2_ is corresponding to the GVD. Higher order dispersions in the fiber have been neglected.

The signal and idler photons are detected by SPDs at both sides with arrival time recorded. Then Alice sends the single photon events to Bob. Time discriminated coincidence measurement can be carried out at Bob side. It can be analyzed by the second-order Glauber correlation function *G*^(2)^. Under the assumption that the temporal dispersion introduced at Bob side is large enough^27^ (Supplementary Information), the *G*^(2)^ function can be expressed as


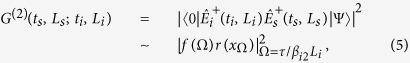


where 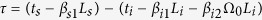.

According to Eq. (5), the coincidence measurement result has the profile of the spectrum of the biphoton state, which is modulated by the reflectivity pattern along the illuminating line in a nonlocal way^27^,^28^. Hence, the reflectivity pattern *r*(*x*_Ω_) can be extracted from the coincidence measurement result. In this scheme, the signal photons are detected without discriminating their frequency and arrival time, hence, the reflectivity pattern along the illuminating line can not be recovered by Alice side only. On the other hand, the idler photons are discriminated according to their frequencies by the arrival time, supporting the nonlocal imaging process in the way of QGI.

The principle can be explained by the transformation of quantum correlation in this scheme. At Alice side, signal photons with different frequencies are mapped to different illuminating positions on the object due to spatial dispersion component. While, at Bob side, the idler photons with different frequencies have different arrival time due to the GVD of the fibers. Hence, the frequency correlation in the photon pairs is transformed to the correlation between the illuminating positions of signal photons on the object and the arrival time of idler photons. Compared with QGI schemes based on momentum-momentum or position-position correlation^3^, our scheme realizes QGI in the time domain thanks to the specific correlation. Since the telecom-band frequency correlated photon pairs can be easily distributed over optical fiber, this scheme provides a practical way to realize long-distance QGI.

### Generation of frequency-correlated photon pairs

**Figure 1:**
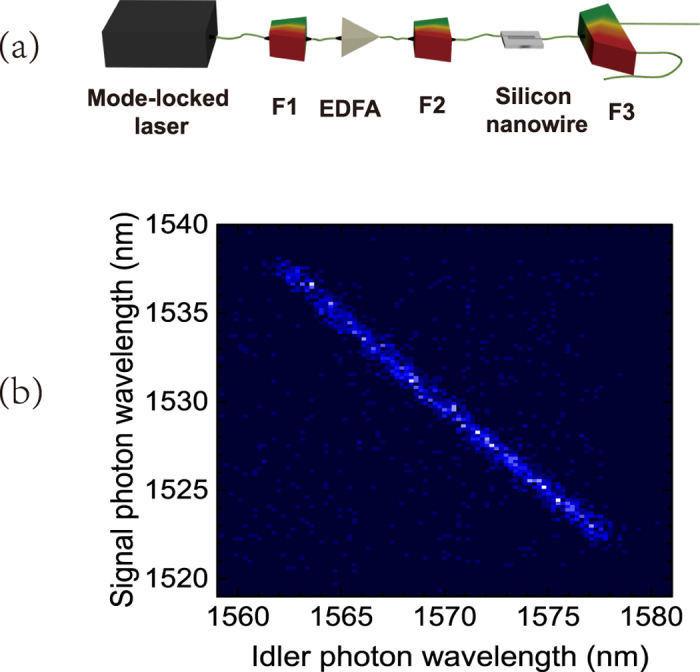
(**a**) The frequency correlated photon-pair source based on the SFWM process in a silicon nanowire waveguide. F1, F2 and F3 are optical filters for pump and signal/idler photons. EDFA: Erbium doped fiber amplifier. (**b**) The joint spectral density of the biphoton state.

In the experiment, the frequency correlated photon pairs are generated by the SFWM process in a silicon nanowire waveguide^24^. The sketch of the source is shown in Fig. 1(a). The pulsed pump light has a center wavelength of 1550.92 nm, a pulse width of about 3.7 ps and a repetitive rate of 40 MHz. It is generated by a mode locked laser, then a filter (F1) is used to confine its spectrum and extend its pulse width. An erbium doped fiber amplifier (EDFA) is used to increase the pump power and the filter (F2) after the EDFA is used to suppress the amplified spontaneous emission at the signal and idler wavelengths. The filters F1 and F2 are composed of dense wavelength division multiplexing devices (DWDMs). The pump light is injected into a silicon nanowire waveguide (11 mm in length, with a cross section of 500 nm × 220 nm) through a lensed fiber. Thanks to the high nonlinearity and the designed low dispersion property of the silicon nanowire waveguide, broad-band frequency correlated photon-pairs can be generated. The signal and idler photons are separated by a filter system (F3) composed of cascaded coarse wavelength division multiplexing devices (CWDMs). The center wavelengths of signal and idler photons are *λ*_*s*0_ = 1530 nm and *λ*_*i*0_ = 1570 nm, respectively.

The joint spectrum density (JSD) of the biphoton state is measured by the method shown in ref. 20. The signal and idler photons are dispersed temporally by a piece of 50 km-long SMF. The measured JSD is shown in Fig. 1(b). It is shown that the wavelengths of signal and idler photons are anti-correlated. The wavelength spans of the signal and idler photons are 16 nm, which are determined by the bandwidth of the filters F3.

### Temporal QGI over 50-km SMF

**Figure 2:**
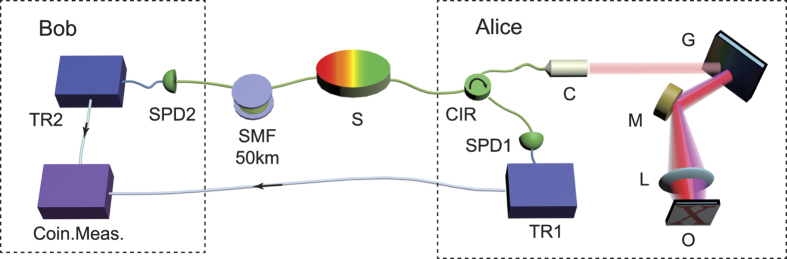
Experiment setup. The frequency correlated photon-pair source (S) has been shown in Fig. 1. The signal photons are distributed to Alice through a short piece of SMF and to Bob through 50 km-long SMF. At Alice side, the signal photons are collimated to a spatial optical beam by a collimator (C) after an optical fiber circulator (CIR), then spatially dispersed by a grating (G), and illuminate the object (O) after a focal lens (L). At Bob side, the GVD of the transmission fiber is utilized to realize temporal dispersion. At both sides, photons are detected by SPDs (SPD1 & SPD2), and the time of single photon events are recorded(time recorder, TR1 & TR2), and coincidence measurement (Coin. Meas.) is carried out. M: mirror.

The sketch of the setup for long-distance temporal QGI is shown in Fig. 2. The source shown in Fig. 1 provides frequency correlated photon pairs. The signal photons are sent to Alice by a short piece of SMF. At Alice side, the signal photons are delivered to an spectrally encoded confocal microscopy (SECM)^29^,^30^ system through a circulator (CIR). In the SECM configuration, the photons are collimated to a spatial optical beam with a 1/*e*^2^ diameter of 2.1 mm. A reflective diffraction grating (600 Line/mm, blaze wavelength equal to 1600 nm) is used to disperse the signal photons spatially. After a lens with a focal length *l* = 25.4 mm, the signal photons are focused on the surface of the object along a line, i.e., the illuminating line, with a length of about 250 μm. The signal photons with different frequencies would be focused on different positions along the illuminating line. The object is a standard photolithographic mask, which is a silica plate with a patterned chrome layer. It provides a reflectivity pattern. The signal photons are partially reflected according the reflectivity pattern along the illuminating line, resulting in modulated spectrum. The reflected signal photons are collected by the collimator and sent to a SPD (ID220, ID Quantique) through the CIR. The single-photon events are recorded with high time-resolution using a time recoder (TR1) in a photon correlator (DPC 230, Becker & Hickl GmbH).

While, the idler photons are sent to Bob through 50 km-long SMF. Then the idler photons are detected by the other SPD and the single photon events are also recorded with high time-resolution (TR2). On one hand, the frequencies of the idler photons are preserved after the propagation through such a long fiber, hence, the long distance distribution of the frequency correlated biphoton states is realized. On the other hand, the transmission fiber provides a GVD of *d* = 900 ps/nm (estimated by the group velocity dispersion parameter of SMF). Since the bandwidth of idler photon is 16 nm, the idler photons are temporally broadened to 15.4 ns.

**Figure 3:**
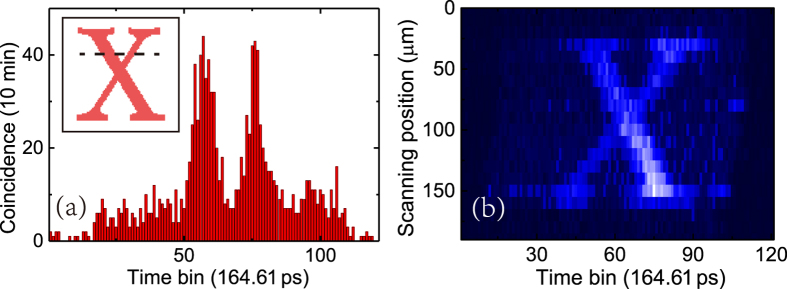
QGI of the object. (**a**) A typical coincidence histogram. The inset is the reflectivity pattern of the object and the dashed line is the illuminating line corresponding to the coincidence histogram. (**b**) The image obtained by step-moving the object with a step of 10 μm.

When sufficient single photon events are collected at Alice and Bob sides, Alice should send the records of the detected signal photons to Bob by a classic channel, and Bob could realize coincidence measurement utilizing recorded signal and idler photon events. In the experiment, the single photon events at both sides are recorded by two channels of the photon correlator with a resolution of 164.61 ps, and the coincidence is calculated by a computer according to the records. A typical measurement result is shown in Fig. 3(a), while the inset shows the reflectivity pattern of the object. The dimension of the pattern is 150 μm × 140 μm. The red areas are the regions with high reflectivity. The dashed line is the illuminating line corresponding to the histogram. It can be seen that the histogram has the shape of the pattern along the illuminating line clearly.

By step-moving the object along the direction orthogonal to the illuminating line, two-dimensional imaging of the object can be obtained, which is shown in Fig. 3(b). The moving step is 10 μm. The measurement time for each illuminating line is 10 minutes. The coincidence counts in every time bin are indicated by different colors. It can be seen that a clear image of the object has been obtained. Considering the long distance between Alice and Bob, this temporal QGI scheme realizes a function, of long distance image transmission like a “quantum faxing machine”. It is worth noting that in the experiment the image generated by the temporal QGI has a little distortion. It is due to the fiber length variation when the temperature changes during the measurement. Since the fiber is as long as 50 km, a temperature variation of 1 °C would lead to an arrival time variation of several nanoseconds for the idler photons traveling through the fiber.

### Resolution

**Figure 4:**
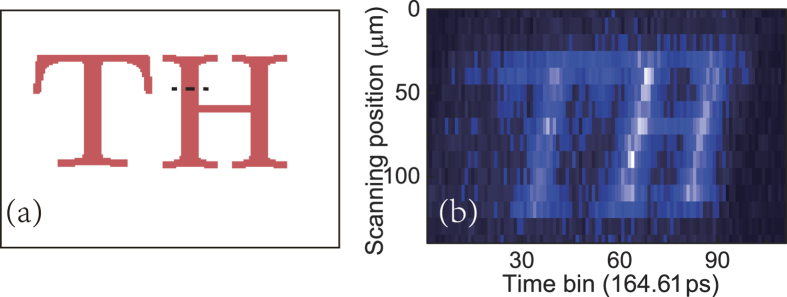
QGI using a object with narrower line width. (**a**) The reflectivity pattern of the target object. The line width is 13 µm at the dashed line, while the width of the image at the corresponding position is 25.6 µm. (**b**) The image generated by the temporal QGI experiment.

To analyze the resolution of our QGI scheme, another object with narrower line width is used, as shown in Fig. 4(a). The image generated by the temporal QGI experiment is shown in Fig. 4(b). The line width of the object is 13 μm at the dashed line, while the width of the image at the corresponding position is about 1.65 ns. According to the experiment parameters (Methods section), it is related to a line width of 25.6 μm, much wider than the actual width. The broadening of the line is due to the limited resolution of the experiment, which is mainly determined by the wavelength resolving power of the grating at Alice side and the timing jitters of the single photon detecting system (including the SPDs and the photon correlator).

Along the illuminating line, a reflectivity point at the object has a point-spread spectral function, which has a full width at half maximum (FWHM) determined by the resolving power of the grating^31^,


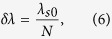


where 
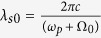
 is the centeral wavelength of signal photons in vaccum, *N* is the total number of grooves in the beam area on the grating. The point spread spectral function will result in a point-spread coincidence peak, with a FWHM of


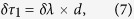


where *d* is the temporal dispersion at Bob side. On the other hand, the impact of the single photon detecting system is denoted by *δτ*_2_, including the effect of timing jitters of two SPDs and the time resolution of photon correlator. Hence, the resolution of the temporal QGI can be estimated by


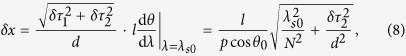


where, *θ*_0_ is the diffraction angle of the signal photons with wavelength *λ*_*s*0_, *p* is the period of the grating. The relation *p*(sin *θ* − sin *θ*_*i*_) = *λ* between the diffraction angle *θ* of the signal photons, the wavelength *λ* and the incident angle *θ*_*i*_, has been used in the equation.

According to the parameters of the experiment setup (*l* = 0.0254 μm, *p* = 1.67 μm, *θ*_0_ = 11.9°, *λ*_*s*0_ = 1530 nm, *N* = 1044 grooves, *δτ*_2_ = 389 ps, *d* = 900 ps/nm), the calculated spatial resolution is about 23.8 μm, agrees well with the experiment results considering the fact that the reflectivity of the object has a rectangluar profile.

Equation (7) shows that the spatial resolution could be improved through (1) increasing temporal dispersion at Bob side; (2) improving the time resolution of the single photon detection system; (3) increasing the beam diameter at Alice side; (4) utilizing an objective lens with smaller focal length at Alice side. Our recent work^23^ shows that *τ*_2_ = 80 ps can be realized in the single photon detection system composed of super conducting nanowire single photon detectors (timing jitter ~60 ps) and high performance photon correlator (timing jitter ~12 ps). If the diameter of the signal photon beam is expended to 5 mm and an objective lens with a focal length of 5 mm is used, a resolution as high as 1.1 μm can be expected.

## Discussion

As a conclusion, we proposed a scheme of temporal QGI. By introducing the temporal dispersion and spatial dispersion on the two photons with frequency correlation respectively, the frequency correlation is transformed to the correlation of arrival time and propagation direction, which is used to realize QGI in time domain. Since the frequency is a stable DOF of photons traveling in optical fibers, it provides a way to realize long distance QGI over optical fibers. It is demonstrated by the experiment with 50-km long SMF, in which the 2-dimensional QGI is realized by step-moving the object like a “quantum fax machine”. The spatial resolution of this scheme is analyzed, showing that it could be less than the wavelength of the illuminating photons by proper system design. This scheme overcomes the obstacle on realizing QGI over large geographical scale, which may inspire new applications in quantum engineering.

## Methods

### Scale transformation relation between the object and the time-domain image

This section discusses the relation between the scale of the object and the image. It is clear that in the direction orthogonal to the illumiating line, the scale of the image should be equal to the scale of the object. In the following, we focus on the scale transformation relation in the direction of the illuminating line.

At Alice side, the relation between the illuminating position *x*_Ω_ on the object and the signal photon frequency *ω*_*p*_ + Ω can be expressed as


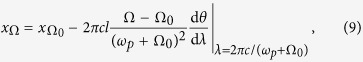


where 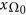 is the illuminating position of signal photons with frequency *ω*_*p*_ + Ω_0_ (the central frequency of the signal photons), *l* is the focal length of the lens, *c* is the velocity of light in vacuum, *θ* is the diffraction angle of light beams after grating, and *λ* is the wavelength of signal photons, which is corresponding to the frequency by the relation *λ* = 2*πc*/(*ω*_*p*_ + Ω). As we use the first order of interference of the grating, the angle of the incident light *θ*_*i*_, the angle *θ* of the diffration light with wavelength *λ*, must satisfy the equation^31^, 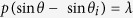, where *p* is the period of the grating. Hence,


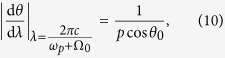


where, *θ*_0_ is the diffraction angle of signal photons with frequency *ω*_*p*_ + Ω_0_. Two points with high reflectivity on the object with distance Δ*x* will reflect signal photons with frequency differency equal to


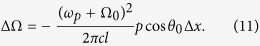


where, *θ*_0_ is the diffraction angle of signal photons with frequency *ω*_*p*_ + Ω_0_.

According Eq. (9), the time differency between the two coincidence peaks resulted from the two high-reflectivity points will be


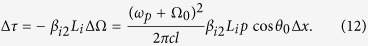


Considering the relation between the dispersion parameter of *d* and *β*_*i*2_, i.e. 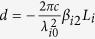, 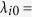 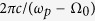
is the central wavelength of idler photons, equation (12) can be expressed using the dispersion parameter *d* as


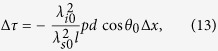


where *λ*_*s*0_ is the central wavelength of signal photons, and *λ*_*s*0_ = 2*πc*/(*ω*_*p*_ + Ω_0_).

## Additional Information

**How to cite this article**: Dong, S. *et al*. Long-distance temporal quantum ghost imaging over optical fibers. *Sci. Rep*. **6**, 26022; doi: 10.1038/srep26022 (2016).

## Supplementary Material

Supplementary Information
