# Supplementary material for: Long-distance temporal quantum ghost imaging over optical fibers
Source: Sci Rep. 2016 May 19;6:26022. doi: 10.1038/srep26022 (PMC4872159; doi:10.1038/srep26022)
Supplement: Supplementary Information [file srep26022-s1.pdf]

Supplementary Material to "Long-distance temporal quantum ghost imaging over optical fibers "

Shuai Dong[1], Wei Zhang[1*], Yidong Huang[1], and Jiangde Peng[1]

[1]Tsinghua National Laboratory for Information Science and Technology, Department of Electronic Engineering, Tsinghua University, Beijing, 100084, China
[*]Corresponding author: zwei@tsinghua.edu.cn

November 11, 2015

Calculation of the $G^{(2)}$ function

It is well known that for biphoton state $|\Psi\rangle$, the second-order Glauber correlation function $G^{(2)}$ can be calculated using [1]

$$G^{(2)}(t_s, L_s; t_i, L_i) = \left|\langle 0|\hat{E}_s^+(t_s, L_s)\hat{E}_i^+(t_i, L_i)|\Psi\rangle\right|^2, \tag{S1}$$

where t_s, t_i is the detection time of signal and ider photons, L_s, L_i is the optical path of signal and idler photons, respectively. Including the dispersion of the transmission fiber, phases will be introduced to the positive-frequency operators, as shown in the Eq.(2) and Eq.(3), following which $\langle 0|\hat{E}_s^+(t_s, L_s)\hat{E}_i^+(t_i, L_i)|\Psi\rangle$ can be calculated with the state in Eq.(1),

$$\begin{aligned}
&\langle 0|\hat{E}_s^+(t_s, L_s)\hat{E}_i^+(t_i, L_i)|\Psi\rangle \\
\sim &\iiint \mathrm{d}\omega_s \mathrm{d}\omega_i \mathrm{d}\Omega f(\Omega) r(x_\Omega) e^{j(\omega_s t_s - \beta_s L_s) + j(\omega_i t_i - \beta_i L_i)} \\
&\times \langle 0|a_s(\omega_s)|\omega_p + \Omega\rangle_s \langle 0|a_i(\omega_i)|\omega_p - \Omega\rangle_i && \text{(S2)} \\
= &\iiint \mathrm{d}\omega_s \mathrm{d}\omega_i \mathrm{d}\Omega f(\Omega) r(x_\Omega) e^{j(\omega_s t_s - \beta_s L_s) + j(\omega_i t_i - \beta_i L_i)} \\
&\times \delta(\omega_p + \Omega - \omega_s)\delta(\omega_p - \Omega - \omega_i) && \text{(S3)} \\
= &e^{j\varphi} \int \mathrm{d}\Omega f(\Omega) r(x_\Omega) e^{j\Omega\tau - j\beta_{i2}\Omega^2/2} && \text{(S4)} \\
= &2\pi e^{j\varphi} \mathcal{F}_\tau(f(\Omega) r(x_\Omega)) * \mathcal{F}_\tau(e^{-j\beta_{i2}\Omega^2/2}), && \text{(S5)}
\end{aligned}$$

where we used the phase coefficient expandation in the Eq.(2) and Eq.(4) and the commutation relation $[a_m(\omega_1), a_n^\dagger(\omega_2)] = \delta_{m,n}\delta(\omega_1 - \omega_2)$, $m, n \in \{s, i\}$, $\delta_{m,n}$ is the Kronecker

delta function, and $\delta(\cdot)$ is the Dirac delta function. $e^{j\varphi}$ includes all the Ω-independent phases, and $\tau = (t_s - \beta_{s1}L_s) - (t_i - \beta_{i1}L_i - \beta_{i2}\Omega_0 L_i)$. $\mathcal{F}_\tau(\cdot)$ denotes the inverse Fourier transformation, as

$$\mathcal{F}_\tau(f(\Omega)r(x_\Omega)) = \frac{1}{2\pi}\int \mathrm{d}\Omega f(\Omega)r(x_\Omega)e^{j\Omega\tau}, \tag{S6}$$

$$\begin{aligned}\mathcal{F}_\tau(e^{-j\beta_{i2}\Omega^2/2}) &= \frac{1}{2\pi}\int \mathrm{d}\Omega e^{-j\beta_{i2}\Omega^2/2}e^{j\Omega\tau} \\ &= \frac{1}{\sqrt{2\pi j\beta_2 L_i}}e^{-j\tau^2/2\beta_{i2}L_i}.\end{aligned} \tag{S7}$$

Combining equation (S5), (S6) and (S7), we obtain

$$\langle 0|\hat{E}_s^+(t_s,L_s)\hat{E}_i^+(t_i,L_i)|\Psi\rangle$$

$$\sim \int \mathrm{d}\tau_1 \left\{\int \mathrm{d}\Omega f(\Omega)r(x_\Omega)e^{j\Omega\tau_1}\right\} e^{-j(\tau-\tau_1)^2/2\beta_{2i}L_i} \tag{S8}$$

$$\sim \int \mathrm{d}\tau_1 \left\{\int \mathrm{d}\Omega f(\Omega)r(x_\Omega)e^{j\Omega\tau_1}\right\} e^{j\frac{\tau\tau_1}{\beta_{i2}L_i}-j\frac{\tau_1^2}{2\beta_{i2}L_i}} \tag{S9}$$

$$\sim f(\Omega)r(x_\Omega)|_{\Omega=\frac{\tau}{\beta_{i2}L_i}}. \tag{S10}$$

From Eq.(S9) to Eq.(S10), we have assumed that a very large dispersion has been introduced at Bob side, so that $\beta_{i2}L_i$ is much larger than the temporal width of idler-photon wavepackets, and neglected the term $\frac{\tau_1^2}{2\beta_{i2}L_i}$, because $\frac{\tau_1^2}{2\beta_{i2}L_i} \ll 1$ [2] .

Lingking Eq.(S1) and Eq.(S10) for clearity, we can get the expression of $G^{(2)}$ function when large temporal dispersion is introduced at Bob side,

$$G^{(2)}(t_s,L_s;t_i,L_i) \sim |f(\Omega)r(x_\Omega)|^2_{\Omega=\tau/\beta_{i2}L_i}, \tag{S11}$$

where $\tau = (t_s - \beta_{s1}L_s) - (t_i - \beta_{i1}L_i - \beta_{i2}\Omega_0 L_i)$.

According to Eq.(S11), on one hand, it is indicated that the coincidence measurement results have the shape of the spectrum of the biphoton state, which is nonlocally modulated by the reflectivity pattern of the object, so by extracting the reflectivity spectra $r(x_\Omega)$, the image of the object can be reconstructed. On the other hand, it can be seen that, after temporal dispersion, the coincidence peak is spread to have a width of $\tau_w = \beta_{i2}L_i\Omega_w$, where Ω_w is the spectrum width of the biphoton state. The spreading of the coincidence peak is due to the GVD introduced at Bob side. For idler photons, using the expandation of the phase coefficient in Eq.(4), the group velocity of idler photons can be expressed as [2]

$$\frac{1}{v_{ig}} = \frac{\partial\beta_i}{\partial\omega_i} = -\frac{\partial\beta_i}{\partial\Omega} = \beta_{i1} + \beta_{i2}(\Omega_0 - \Omega). \tag{S12}$$

So the time needed for idler photons from the source to the SPD at Bob side will be

$$t_\Omega = \frac{L_i}{v_{ig}} = \beta_{i1}L_i + \beta_{i2}L_i(\Omega_0 - \Omega). \tag{S13}$$

For idler photons with frequency difference $\Delta\Omega$, the time delay difference will be

$$\Delta t = -\beta_{i2} L_i \Delta\Omega, \tag{S14}$$

which is coincident with the spread of the coincidence peak. In fact, after the spatial dispersion at Alice side, and the temporal dispersion at Bob side, the frequency correlation in the photon pairs is transformed to the correlation between the illuminating position x_Ω of signal photons on the object and the travel time t_Ω of idler photons before detected by the SPD. Compared with previous QGI schemes based on momentum-momentum or position-position correlation, our time domain QGI is realized utilizing this kind of correlation.

References

[1] Y. H. Shih, *An Introduction to Quantum Optics: Photons and Biphoton Physics* (CRC press,2011).

[2] S. A. Akhmanov and S. Y. Nikitin, *Physical Optics* (Clarendon Press, Oxford, 1997).
